# The Impact of Foreign Direct Investment on Urban Green Total Factor Productivity and the Mechanism Test

**DOI:** 10.3390/ijerph191912183

**Published:** 2022-09-26

**Authors:** Mingliang Zhao, Yue Gao, Qing Liu, Wei Sun

**Affiliations:** 1 Department of International Economics and Trade, Shandong University of Finance and Economics, Jinan 250002, China; 2 School of Travel Agency Management, Shandong College of Tourism and Hospitality, Jinan 250200, China; 3 Key Laboratory of Regional Sustainable Development Modeling, Institute of Geographic Sciences and Natural Resources Research, Chinese Academy of Sciences, Beijing 100101, China; 4 College of Resources and Environment, University of Chinese Academy of Sciences, Beijing 100049, China

**Keywords:** open economic transformation, green economic development, scientific and technological innovation, industrial structure upgrading

## Abstract

This paper employs the slack-based model directional distance function to measure the green total factor productivity of each city, using the panel data of 284 prefecture-level cities in China from 2004 to 2019 and considering the unexpected output. The results are as follows: ① Foreign direct investment significantly suppresses the improvement of urban green total factor productivity, and the negative impact on the green technology progress index is the main reason to inhibit the increase of the green total factor productivity. The results are still significant through a series of robustness tests such as replacing variables and eliminating outliers; the positive intermediary effect of scientific and technological innovation exists, and the Sobel test and bootstrap random sampling test are passed. The upgrading of industrial structure has a positive regulating effect on the improvement of urban green total factor productivity. ② The impact of foreign direct investment on urban green total factor productivity has regional heterogeneity. The inhibitory effect of foreign direct investment on resource-based cities and non-coastal cities is greater than that on non-resource-based cities and coastal cities, and the negative impact on China-Europe train opening cities is greater than that on non-opening cities. Accordingly, the paper puts forward policy suggestions from the aspects of improving the quality of foreign direct investment and implementing differentiated management.

## 1. Introduction

Since 1993, China’s actual utilization of foreign capital has always been ranked first among developing countries and second in the world. In 2020, China replaced the United States and became the world’s largest recipient of foreign investment [1]. Foreign direct investment (FDI) can provide funds for the economic development of our country to some extent, bring advanced technology and management experience, cultivate high-quality talents and promote the improvement of productivity [2,3]. However, at the same time, the FDI is mainly focused on industries with high pollution and emission. Long-term extensive investment production has brought about problems in our country such as excessive energy consumption, environmental pollution and an imbalance of the economic structure. Since the beginning of the 21st century, China’s economic structure and the principal contradiction in society have changed. With its strategic, programmatic and guiding nature, the “Five Development Concepts” have pointed out the direction and focus of China’s development. Green and high-quality development has become a national strategy. Especially under the new pattern of “dual circulation” development, the government should abandon the development concept of “seeking foreign capital quantity unduly” and establish the concept of “taking count to quantity and quality simultaneously”. Green is the background color of the new development concept, which solves the problem of coordination between economic development and natural environmental protection [4]. Opening-up is the general trend of development, which is the guide to action for China’s all-round and high-level opening-up. The FDI creates conditions for technology transfer and spillover of transnational corporations, Chinese enterprises should further absorb the advanced green innovative technologies of foreign countries. As an endogenous driving force for sustainable and high-quality economic development, independent technological innovation can break technological dependence and realize technological localization, which can improve the production mode with high pollution and high emissions, provide a guarantee for the green development of the economy and promote the realization of the goal of carbon peaking and carbon neutrality [5]. In the process of opening up and development, the industrial structure is also constantly optimized and upgraded, providing impetus for green and high-quality development. At the same time, the implementation of the Belt and Road Initiative and the opening of the China-Europe Railway Express have improved the layout of China’s opening up to the outside world and attracted a number of high-quality foreign enterprises to China, which provided more opportunities for economic development [6]. This paper will focus on analyzing the impact of FDI on green total factor productivity and the potential impact mechanism of scientific and technological innovation and industrial structure optimization from the city level and investigate the heterogeneous impact of FDI from several aspects such as coastal opening, opening of the China-Europe Railway Express and transformation of resource-based cities. Considering the undesired output, the slack-based model (SBM) directional distance function is used to measure the green total factor productivity of each city. The following research hypotheses are proposed:

In the process of attracting foreign investment, China sacrifices environmental quality and lacks environmental regulation, which is not conducive to the development of a green economy. In addition, with its advanced management experience and technical advantages, foreign capital reduces the living space of local enterprises in the market and the ability to participate in competition and innovation, and to a certain extent, inhibits the development of regional green economy.

**Hypothesis** **1.** *Foreign direct investment has an inhibitory effect on urban green total factor productivity*.

The innovation of science and technology is leading the first driving force of the development of the green economy; the host country, by imitating and absorbing advanced foreign science and technology and management experience, can promote the technology spillover effect. Independent technological innovation can be optimized for the production of technology, which can improve the level of pollution control technology and reduce pollution emissions and energy consumption, in order to promote green total factor productivity.

**Hypothesis** **2.** *Scientific and technological innovation can play a mediating effect to solve the disharmony between foreign direct investment and green total factor productivity.*

With the transformation and upgrading of the industrial structure, the proportion of green industries with low emissions and high added value is constantly increasing, which promotes the green development of traditional resource-based industries. In the process of industrial optimization and upgrading, an efficient, clean and recyclable green production system is gradually constructed to promote green and sustainable economic development.

**Hypothesis** **3.** *The transformation and upgrading of the industrial structure play a positive moderating role in the effect of foreign direct investment on green total factor productivity.*

This paper expands the analysis from the following dimensions: First, it analyzes the direct effect of FDI on green total factor productivity, and further tests the differences in the sources of impact effects from the two aspects of green technology efficiency and green technology progress. Secondly, the mechanism of FDI affecting green total factor productivity is discussed in detail, and the indirect effect of scientific and technological innovation as a mediating variable is tested. Considering that the upgrading process of the industrial structure is relatively slow and the influence result has hysteresis, we tested whether there is a moderating effect. Finally, considering the differentiated impact of urban resource endowment and national policy guidance in the process of opening up, the paper examines the impact of FDI on the green total factor productivity of different types of cities, and comprehensively and carefully discusses the relationship, influence mechanism and differentiated impact between them, which is of great practical significance for the country to optimize the layout of opening up and further accelerate the transformation of green economic development.

## 2. Literature Review and Influencing Mechanism Analysis

With the increase of FDI in developing countries, the international community has paid continuous attention to environmental issues and the need for green transformation and development in developing countries. The relationship between foreign direct investment and green total factor productivity has been discussed in detail. However, due to the differences in research methods, hypotheses and time intervals, there is no consensus at present. Some scholars believe that the technology spillover effect and learning effect brought by FDI can bring advanced foreign management experience and science and technology, significantly improve the knowledge and skill level of local personnel, promote the optimization of industrial structure and process reform, and form the “pollution halo effect” [7,8,9,10]. You and Xiao [11] believe that FDI has brought standardized production processes and technologies to host countries, improved energy utilization efficiency and played a role in protecting local ecological environment. Eskeland and Harrison [12] found that foreign factories usually use clean energy instead of traditional “dirty fuel”, which promotes the development of a green economy in host countries. From the perspective of manufacturing industry, Li et al. [13] found that FDI had a significant promoting effect on the green total factor productivity of the manufacturing industry, in which the passing threshold effect of human capital and environmental regulation played an important role. However, there is the theory of a “pollution paradise”, believing that developed countries usually export highly polluting and energy-intensive industries to developing countries by means of foreign investment, which significantly inhibits the development of a green economy in host countries. Chen et al. [14] believe that China has made concessions in environmental protection in order to attract a large number of foreign investors. Foreign-funded enterprises pay reduced environmental taxes when they have their production in China, which causes damage to the ecological environment. D ‘Agostino and Lorena [15] found that under the pressure of global competition, transnational corporations would transfer environment-polluting production activities to developing countries with loose environmental regulations, so as to reduce production costs. Wang et al. [16] compared FDI as a double-edged sword, which brings economic growth and technological innovation to the host country while causing serious pollution to the local environment. In recent years, the interaction effect of foreign direct investment and other variables on green total factor productivity has been explored. Wang et al. [17] found that the interaction between environmental regulation and FDI promoted the growth of green total factor productivity in prefecture-level cities in China. Hang and Ren [18] found that reasonable interaction between FDI and industrial structure optimization can positively regulate green total factor productivity. FDI not only increases capital supply, but also promotes technological progress and industrial structure upgrading, which effectively drives economic growth. From the micro perspective, Wu et al. [19] believe that green innovation has an obvious promoting effect on the green total factor productivity of enterprises. Furthermore, this effect is based on the heterogeneity of different enterprise characteristics and patent types. At the industrial level, Sun et al. [20] found that green technology progress in emerging strategic industries can reduce production costs, improve the utilization rate of natural resources and improve ecological efficiency. Zhang et al. [21] found that ecological innovation promoted the development of green industry in China by studying the industrial green total productivity of 30 provinces and cities in China. From the perspective of the chemical industry, Yang et al. [22] found that technological progress promoted the sustainable development of this industry by reducing carbon dioxide emissions and improving capital saving performance. From the perspective of cities, Fan et al. [23] found that the innovation level of green technology promoted the development of green economy in Chinese cities, and this conclusion was also verified in the development of Italian cities [24]. Mao et al. [25] studied cities in the Yellow River basin using the tobit model and found that green technology progress drove the increase of green total factor productivity in the whole region, but the upward trend of each basin is different, and this conclusion is also applicable to the Yangtze River basin [26]. Based on the analysis of China’s carbon emission trading policy, Huang and Huang [27] found that the implementation of this policy promoted the green total factor productivity of pilot cities, which was mainly achieved by low-carbon innovation. Some scholars discussed the mechanism from the perspective of industrial structure. Ji [28] believed that the rationalization of the industrial structure was an important reason for the improvement of green total factor productivity in 30 provinces of China, but this effect had a lag. For the Yangtze River basin, Sun et al. [29] found that the more economically developed provinces and cities were, the higher the industrial structure was, and the greater the promotion to the green total factor productivity was. From the perspective of carbon emission reduction, Yang et al. [30] found that the rationalization of the industrial structure can significantly inhibit carbon emissions and promote the green ecological development of the cities. Liang et al. [31] found that there is a nonlinear relationship between industrial upgrading and green total factor productivity growth rate through the study of less developed provinces in China. The rationalization of industrial structure has an important impact on this effect.

From the current research, there is still a debate on the relationship between FDI and green total factor productivity. Studies in developed countries mostly support the pollution halo effect, while studies in developing countries mostly support the pollution paradise hypothesis. The discussion on the influencing mechanism is not in-depth enough, and the differential influence of urban resource endowment and national policy orientation is not considered enough. The influence mechanism between the two and the different roles played by different types of cities are rarely analyzed. As a developing country, China should conform to the pollution paradise hypothesis. Based on the panel data of Chinese cities from 2007 to 2019, this paper uses the SBM directional distance function to measure green total factor productivity. This paper analyzes the impact of foreign direct investment on green total factor productivity through econometric models and applies the Sobel test and bootstrap random sampling to test the role of scientific and technological innovation and industrial structure upgrading in this impact.

## 3. Research Methods and Data Description

Green total factor productivity is measured by SBM directional distance function. The econometric model is used to empirically test the effect of foreign direct investment on green total factor productivity, and the Sobel test and bootstrap random sampling are used to test the existence of the mediating effect of scientific and technological innovation, so as to explore the moderating effect of industrial structure upgrading. Then, the impact of regional heterogeneity of cities is investigated.

### 3.1. Research Methods

#### 3.1.1. Measurement of Green Total Factor Productivity

Data Envelopment Analysis (DEA) is a non-parametric analysis method used to evaluate the relative efficiency of decision-making units with multiple inputs and outputs. The Malmquist index is one of the models commonly used to evaluate the efficiency of decision-making units in the DEA method. It is mainly used to measure the change of total factor productivity of decision-making units in different periods [32]. Compared with the measurement method of total factor productivity that only considers traditional input-output variables, the measurement of green total factor productivity includes energy consumption and pollutant emissions. Referring to Charnes et al. [33], on the basis of the directional distance function proposed by Chung et al. [34], this paper uses the non-radial and non-angular SBM directional distance Malmquist–Luenberger index developed by Fare et al. [34,35] to measure the green total factor productivity of 284 prefectural-level cities from 2004 to 2019. The Malmquist–Luenberger formula from *t* to *t* + 1 is

(1) MLtt+1=[1+D0t→(xt,yt,zt;gt)1+D0t→(xt+1,yt+1,zt+1;gt+1)×1+D0t+1→(xt,yt,zt;gt)1+D0t+1→(xt+1,yt+1,zt+1;gt+1)]12

(2) $$MEC_{t}^{t+1}=\frac{1+\vec{D_{0}^{t}}(x^{t},y^{t},z^{t};g^{t})}{1+\vec{D_{0}^{t+1}}(x^{t+1},y^{t+1},z^{t+1};g^{t+1})}$$

(3) $$MTC_{t}^{t+1}=\left[\frac{1+\vec{D_{0}^{t+1}}(x^{t},y^{t},z^{t};g^{t})}{1+\vec{D_{0}^{t}}(x^{t},y^{t},z^{t};g^{t})}\times \frac{1+\vec{D_{0}^{t+1}}(x^{t+1},y^{t+1},z^{t+1};g^{t+1})}{1+\vec{D_{0}^{t}}(x^{t+1},y^{t+1},z^{t+1};g^{t+1})}\right]$$

where $\vec{D}$ is the directional distance function, $x^{t},y^{t},z^{t}$ are the input index, desired output and undesired output, respectively. $g^{t}$ is the direction vector. In this paper, it is set as *g* = (−*x*, *y*, −*z*), which means to achieve the sustainable production goal, ensure the maximization of expected output, and minimize factor input and undesired output.

$ML_{t}^{t+1}=MEC_{t}^{t+1}\times MTC_{t}^{t+1},\mathrm{where}\;MEC_{t}^{t+1}$ is the green technology efficiency, which measures the catch-up degree of each decision-making unit to the production possibility frontier from *t* to *t* + 1 and represents the output growth stimulated by internal efficiency changes. If the value of this index is greater than 1, it means that the green technology efficiency in period *t* + 1 has been improved compared with that in period *t*. $MTC_{t}^{t+1}$ represents the level of green technology, which measures the convergence degree of each decision-making unit to the optimal production frontier from *t* to *t* + 1 and represents the output growth caused by technological progress. If the value of this index is greater than 1, it means that there is a new breakthrough in green technology during the period from t to *t* + 1. Since the undesired output SBM-ML index reflects the change rate of green total factor productivity between two periods in a region, this paper refers to the practice of Cai and Zhou [36], takes 2004 as the base period, sets the green total factor productivity of this year as 1, and uses continuous multiplication to calculate the green total factor productivity of each year in a sequential way. The same treatment is applied to the technical progress index and technical efficiency index.

The input-output indicators of green total factor productivity are shown in Table 1. Among them, the physical capital stock is measured by the “perpetual inventory method” according to Zhang Jun [37], and the formula is *K_it_* = *I_it_* + (1 − δ*_it_*) *k*_*it*−1_, where *i* represents each city, *t* represents the year, *K, I* and *δ* represent capital stock, investment and depreciation rate, and the depreciation rate *δ* is set at 9.6%. At the same time, taking 2004 as the base period, the fixed asset investment price index is used to eliminate the influence of price changes. The calculation formula of base period capital stock is *K_it_* = *I_it_*/(*g* + *δ*), and *g* is the annual growth rate of fixed asset investment from 2004 to 2019. This paper uses the Matlab software program to calculate *ML, MEC* and *MTC* values.

**Figure 1:**
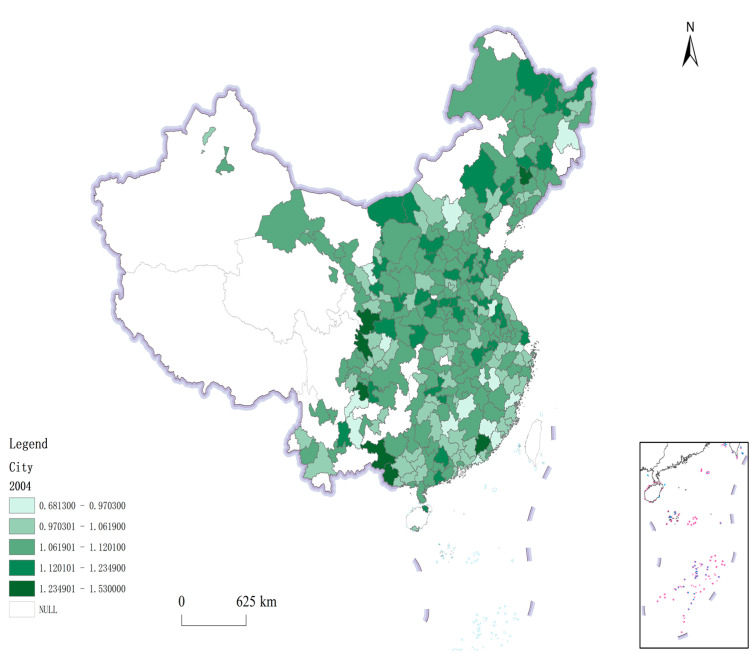
ML index (2004).

**Figure 2:**
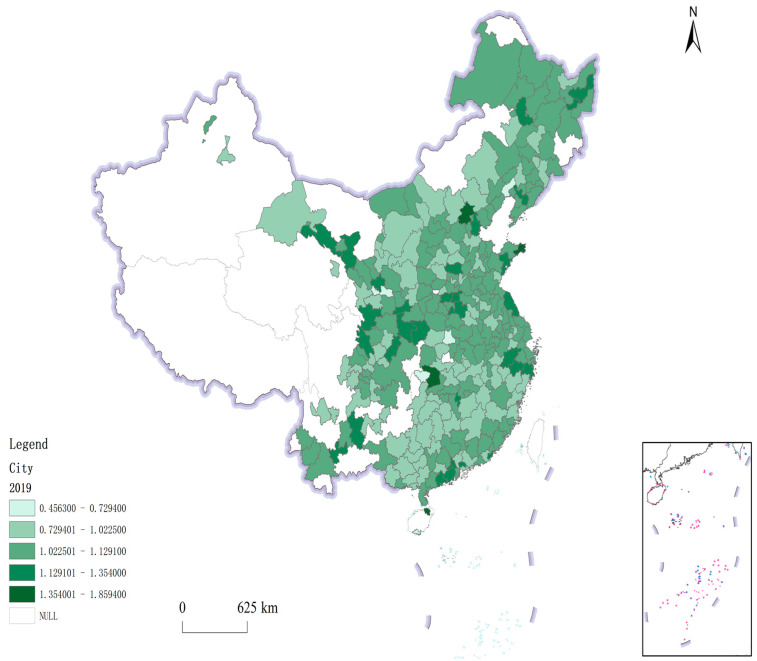
ML index (2019).

Figure 1 and Figure 2 respectively, show the green total factor productivity of 284 sample cities in 2004 and 2019. Through comparison, it can be found that the green total factor productivity of the sample cities in 2004 is relatively average, and the gap is small. By 2019, the gap of green total factor productivity between cities had gradually widened. From the perspective of the characteristics of the level of green total factor productivity, Beijing, Weihai, Qingdao, Shanghai, Zhengzhou and other cities ranked the top in China’s green total factor productivity in 2019, mainly concentrated in coastal cities, regional central cities and some cities that enjoy more favorable national policies. The green total factor productivity values of resource-based cities represented by Karamay, Baise, Shuangyashan, Chifeng and Anshan are all located in the downstream of the sample cities. It is necessary to test the impact of FDI on green total factor productivity in these different types of cities in Chapter 4 to clarify whether the improvement of green total factor productivity is driven by FDI or affected by environmental regulations and other factors, so as to provide guidance for the development of cities with low green total factor productivity.

#### 3.1.2. Model Specification

According to the literature review and theoretical analysis, besides FDI, the factors affecting green total factor productivity also include resource abundance and environmental regulation and other factors. Therefore, based on the theoretical model of Hulten [38], the model is extended according to the research needs. Assuming that the urban production function is of the C-D function form, the standard C-D production function can be extended to:

(4) Y=A(FDI,RES,ER,INF,GOV,t)F(K,L,E)

where *Y* represents the urban output level; *FDI* means FDI; *RES* is resource abundance; ER is environmental regulation; *INF* refers to infrastructure construction; *GOV* is the degree of government intervention; *K* is capital factor input; *L* is labor factor input; *E* represents energy factor input. *A(∙)* represents the Hicks-neutral technology progress function, which is composed of multivariate combinations. *A*(∙) can be expressed as:

(5) $$A(FDI_{it},RES_{it},ER_{it},INF_{it},GOV_{it})=A_{i0}e^{\lambda_{i}t}FDI_{it}^{\alpha_{i}}RES_{it}^{\beta_{i}}ER_{it}^{\varphi_{i}}INF_{it}^{\delta_{i}}GOV_{it}^{\gamma_{i}}$$

Introducing Equation (5) into Equation (4), we can get:

(6) $$Y_{it}=A_{i0}e^{\lambda_{i}t}FDI_{it}^{\alpha_{i}}RES_{it}^{\beta_{i}}ER_{it}^{\varphi }INF_{it}^{\delta_{i}}GOV_{it}^{\gamma_{i}}\cdot F(K_{it},L_{it},E_{it})$$

where *A*_*i*0_ is the initial production technical efficiency level; *λ* is exogenous production technology change; *α, β, φ, δ, γ* are the elastic coefficients of FDI, resource abundance, environmental regulation, infrastructure construction and government intervention degree; *i* stands for city; t stands for time. The calculation formula can be obtained by dividing $F(K_{it},L_{it},E_{it})$ both sides of Equation (7):

(7) $$ML_{it}=\frac{Y_{it}}{F(K_{it},L_{it},E_{it})}=A_{i0}e^{\lambda_{i}t}FDI_{it}^{\alpha_{i}}RES_{it}^{\beta_{i}}ER_{it}^{\varphi_{i}}INF_{it}^{\delta_{i}}GOV_{it}^{\gamma_{i}}$$

Taking the natural logarithm of Equation (7), the panel regression model can be set as follows:

(8) $$\ln ML_{it}=\ln A_{i0}+\lambda_{i}t+\alpha_{i}\ln FDI_{it}^{}+\beta_{i}\ln RES_{it}^{}+\varphi_{i}\ln ER_{it}^{}+\delta_{i}\ln INF_{it}^{}+\gamma_{i}\ln GOV_{it}^{}+\theta_{it}+\pi_{it}$$

where *μ_it_ = θ_it_ + ψ_it_, θ_it_* represents city fixed effect; *ψ_it_* is a time fixed effect; *π_it_* is the random error term.

### 3.2. Description of Variables and Data Sources

#### 3.2.1. Variable Description

The variable description is shown in Table 2. Referring to the expression method of Yang [39], the stock data of *FDI* is selected for expression and converted into *RMB* according to the current exchange rate for calculation. The formula for calculating capital stock is *FDI_it_* = *FDI*_*it*−1_ (1 − *δ*) + *I_it_*, where *FDI_it_* represents the stock of *FDI* in period t of city *i, FDI_it−*1*_* represents the stock of *FDI* in period *t*−1 of city *i*, and *δ* is the depreciation rate, which is set at 15% according to the experience of Hu [40]. The formula for calculating the stock of *FDI* in the base period is *FDI_it_* = *I_it_*/(*G* + *δ*), where *I_it_* represents the *FDI* in period *t* of city *i*, *G* is the annual growth rate of *FDI* from 2004 to 2019, and *δ* is the depreciation rate. In the calculation of environmental regulation (*ER*), the entropy method is used to deal with the discharge of industrial wastewater, industrial sulfur dioxide and industrial smoke dust. For the convenience of later analysis, the inverse is taken to obtain the environmental regulation index of each city.

#### 3.2.2. Data Sources

In this paper, the panel data of 284 cities from 2004 to 2019 were selected for analysis. The data were collected from China Environmental Statistical Yearbook, China Industrial Statistical Yearbook, China Science and Technology Statistical Yearbook, China Energy Statistical Yearbook and the statistical yearbooks of various prefecture-level cities from 2005 to 2020. In order to eliminate the interference of heteroscedasticity and solve the dimensional problem, all variables are logized in this paper.

## 4. Analysis of Research Results 

### 4.1. Correlation Analysis 

For model selection, this paper first conducted a B-P test, and the test results are shown in Table 3. The *p*-value of the B-P test is 0, so it is considered that the model in this paper is not suitable for the mixed regression model. Then, the Hausman test was performed, and the corresponding statistic was 58.21 with a *p*-value of 0, strongly rejecting the original hypothesis. Therefore, the panel fixed effect model should be used for the regression in this paper.

The specific model is set as follows:

(9) $$\ln ML_{it}=\alpha_{0}+\beta_{0}\ln FDI_{it}+\delta X_{it}+\gamma_{i}+\phi_{t}+\varepsilon_{it}$$

(10) $$\ln MEC_{it}=\alpha_{1}+\beta_{1}\ln FDI_{it}+\delta X_{it}+\gamma_{i}+\phi_{t}+\varepsilon_{it}$$

(11) $$\ln MTC_{it}=\alpha_{2}+\beta_{2}\ln FDI_{it}+\delta X_{it}+\gamma_{i}+\phi_{t}+\varepsilon_{it}$$

where *i* and *t* represent prefecture-level cities and years, and *ML_it_*, *MEC_it_* and *MTC_it_* represent green total factor productivity, green technology efficiency index and green technology progress index in turn. *FDI_it_* represents the actual amount of foreign capital utilized by all prefecture-level cities each year, and *X_it_* represents the control variable. *β_0_, β_1_,* and *β_2_* are the coefficients we focus on in this paper. *φ_t_* is the year fixed effect. *γ_i_* is the city fixed effect. *ε_it_* is the random error term to reduce endogenous interference.

Table 4 reports the benchmark regression results of the impact of FDI on urban green total factor productivity. After the introduction of all control variables, FDI has a restraining effect on the improvement of urban green total factor productivity, and it is significant at the 5% level. The reasons may be the following three aspects: First, FDI brings pollution transfer. Developed countries transfer their enterprises with high pollution and high emission to our country and make extensive use of the natural resources, which has caused great damage to the ecological environment and hindered the high-quality economic development. Second, in order to avoid technology diffusion, multinational enterprises will adopt “technology lock-in” to ensure their competitive advantages. Chinese enterprises cannot learn the core content of advanced technology and can only engage in low-technology production work, which prevents technology localization. Moreover, different enterprises have different absorptive capacities of technology. If local enterprises have poor absorptive capacities and large technological potential difference, the technological spillover effect of FDI cannot be brought into play, which weakens the endogenous driving force of economic growth and hinders the green transformation. Third, foreign-funded enterprises can quickly seize the domestic market by virtue of their perfect management experience and advanced sales methods, which greatly occupies the profit margin of local Chinese enterprises. As a result, enterprises are unwilling to use a large amount of funds to carry out high-risk R&D activities, and their willingness to innovate decreases, which is not conducive to high-quality economic development.

From the perspective of the regression results of control variables, the impact of the resources abundance of urban on GTFP is significantly negative effect coefficient. This is because the long-term resource-intensive activities in cities lead to local economic development falling into a single industrial structure dominated by resource consumption. This traditional extensive development with high energy consumption and low income leads to a serious mismatch between economic growth and environmental protection, which has a negative impact on the development of green economy. Infrastructure construction significantly deteriorates local green total factor productivity. The continuous expansion of urban infrastructure occupies local natural and ecological resources and generates a large amount of undesirable output such as solid waste and soot. Moreover, extensive infrastructure construction will cause capital crowding-out effect, which will have a negative impact on local economic development efficiency and ecological environment. The impact of environmental regulation on urban GTFP is positive and significant at the level of 1%, indicating that the strengthening of environmental regulation has effectively suppressed the emission of urban industrial waste, forced enterprises to seek efficient and low emission production mode, and promoted the green development of urban economy. Government intervention is not conducive to the improvement of urban green total factor productivity. The stronger the government intervention is, the more inclined the local government will be to use administrative means to force enterprises to control pollution. All of these cause the distortion of resource allocation and hinder the high-quality development of the economy.

The regression results of decomposition term of GTFP in Table 5 show that the influence of FDI on green technology efficiency level fails to pass the significance test, but it significantly inhibits the progress of green technology at the level of 10%, which indicates that the main reason why FDI inhibits urban GTFP is that it hinders the improvement of green technology. The specific reasons may be the following three aspects: firstly, the ability of independent innovation is poor. In order to realize economic benefits quickly, China has a serious “taking doctrine” of foreign technology and a lack of continuous investment in independent innovation, which is not conducive to the progress of green technology. Second, the motivation for R&D is insufficient, and the incentive mechanism needs to be improved. At present, China’s incentive mechanism is lagging behind, lacking a scientific performance evaluation system and is insufficient in promoting the research and development work. Third, the market is in disorder and the external environment is not ideal. The innovation of science and technology in our country is in the transition stage, and the original rules of the innovation system do not conform to the present development situation anymore, while new rules have not yet been formed. The ambiguity of market system greatly hinders innovation activities and is not conducive to the progress of green technology.

### 4.2. Robustness Analysis

#### 4.2.1. Replace Explanatory Variables

Taking the logarithm of the actual utilized foreign capital stock as the explanatory variable, it may not be able to completely eliminate the potential impact of price factors on urban green total factor productivity. Therefore, this paper replaced the measurement method of explanatory variables with the proportion of actual utilization of foreign capital in GDP of each city every year, so as to eliminate the impact of price level on estimation accuracy. The results are shown in column (1) of Table 6, after replacing the calculation method of explanatory variables, the coefficient of explanatory variables is still negative, which indicates that the negative effect of FDI on urban GTFP is robust.

#### 4.2.2. Replace Control Variables

In the benchmark regression part, resource abundance, environmental regulation, infrastructure construction and government intervention were used as control variables for regression. Considering that different control variables will affect the final results, this paper selects the proportion of education expenditure, financial service level and per capita GDP to replace the original control variables for regression. The regression results are shown in column (2) of Table 6, and the numerical signs and significance of the regression results have not changed significantly compared with those above, which proves that the conclusions of this paper are reliable.

#### 4.2.3. Eliminate Outliers

In order to exclude the possible influence of outliers on the estimation results, the control variables lower than 5% and higher than 95% quantiles were replaced by 5% and 95% quantiles, respectively. The results are shown in column (3) of Table 6. After replacing the outliers, the regression shows that the inhibitory effect of FDI on urban GTFP is significant, which proves that the conclusion is stable.

### 4.3. Influence Mechanism Analysis

Benchmark regression results show that FDI significantly inhibits urban GTFP. In order to further explore the specific mechanism, the intermediary effect is examined from the perspective of scientific and technological innovation based on literature review. Considering that the upgrading process of industrial structure is relatively slow and the influence results have lag, this paper tests whether the industrial structure optimization plays a regulating effect.

#### 4.3.1. Mediating Effect

In this paper, the following mediating effect model is constructed for regression, where mediating variables *RD_it_* represents the stock of science and technology expenditure in the general budget of city *i* at period *t*. Scientific and technological innovation can promote the green development of economy by accelerating the development of clean energy technology and reducing pollutant emission. At present, government expenditure on science and technology plays a large role and has a strong incentive effect on innovation willingness. Considering the obvious cumulative effect in the process of technological development, this paper uses the stock of science and technology expenditure in the general budget of local finance to express it [41], and the stock is calculated according to the calculation method of the stock of FDI.

(12) $$\ln ML_{it}=\alpha_{1}+\beta_{1}\ln FDI_{it}+\delta X_{it}+\gamma_{i}+\phi_{t}+\varepsilon_{it}$$

(13) $$\ln RD_{it}=\alpha_{2}+\beta_{2}\ln FDI_{it}+\delta X_{it}+\gamma_{i}+\phi_{t}+\varepsilon_{it}$$

(14) $$\ln ML_{it}=\alpha_{3}+\beta_{3}\ln FDI_{it}+\beta_{4}\ln RD_{it}+\delta X_{it}+\gamma_{i}+\phi_{t}+\varepsilon_{it}$$

According to the regression results in Table 7, the coefficient of the scientific and technological innovation and FDI is significant. The mediating effect of scientific and technological innovation exists. The action path of: FDI → scientific and technological innovation → GTFP can be verified. In order to further verify the robustness of the above mediating effect test conclusions, the Sobel test and bootstrap random sampling test are adopted. The Sobel test *p*-value is zero, indicating that the intermediary variable passed the Sobel test. Bootstrap test of population random sampling is used to further verify the mediating effect. The bootstrap test is more sensitive to the mediating variable and the final result is more accurate. If the confidence interval of the final result did not include 0, the mediating effect was considered significant. The results of 1000 random sampling are shown in Table 7.

Specifically, column (2) of Table 7 shows that FDI significantly improves the scientific and technological innovation level of cities at the level of 1%, which verifies the “innovation compensation effect” of FDI to a certain extent. There are technology spillovers of FDI in host countries. To further verify whether the mediating effect is complete, science and technology investment and FDI are included in the model for analysis in column (3). Science and technology innovation still plays a significant positive effect, and the regression result is significant at the 1% level. After controlling the indirect effect of science and technology input, the effect of FDI on urban GTFP is still significantly negative at the level of 5%, and the coefficient decreases slightly compared with the results in column (1). This indicates that scientific and technological innovation plays a positive mediating role in this process, but the promoting effect does not fully offset the negative effect of FDI on GTFP growth.

#### 4.3.2. Moderating Effect

With the increase of FDI, the continuous optimization of industrial structure may have a moderating effect on green total factor productivity. In this paper, the ratio of tertiary industry to GDP is selected to represent the upgrading of industrial structure [42], and the interaction term between it and *FDI* is added to the benchmark regression model for verification. The model is set as follows: moderating variables *IS_it_* represents the proportion of tertiary industry in period t of city *i*. Industrial structure can affect economic development by optimizing factor allocation and improving production efficiency. The rise of tertiary industry can replace the traditional inefficient and energy-consuming production mode, which has a positive effect on the transformation of green economy. Therefore, this paper selects the ratio of tertiary industry to GDP to represent the upgrading of industrial structure.

(15) $$\ln ML_{it}=\alpha_{0}+\beta_{0}\ln FDI_{it}+\beta_{1}\ln IS_{it}+\beta_{2}\ln FDI_{it}\times \ln IS_{it}+\delta X_{it}+\gamma_{i}+\phi_{t}+\varepsilon_{it}$$

The results in column (1) and (2) of Table 8 show that the coefficient of FDI is negative, while the coefficient of interaction term is 0.165, the coefficient of core variable being opposite to the coefficient of interaction term, indicating that there is a significant regulatory effect and, the interaction effect of FDI and industrial structure upgrading has a certain positive impact on the growth of green total factor productivity. Giving full play to their transfer and superposition function is beneficial to the green development of sample cities. On the one hand, the introduction of high-quality foreign capital and the transformation and upgrading of industrial structure promote the research and development of new energy technology products gradually eliminate the enterprises with high energy consumption and high pollution. The proportion of green industries with low emission and high added value is increasing in the industrial structure. This promotes the green development of traditional resource-based industries and, the formation of an efficient, clean, recyclable green production system. On the other hand, the inflow of a large amount of foreign capital makes up for the capital scarcity of local enterprises in the development process, so that domestic enterprises change the inefficient production mode through the learning effect and imitation effect, and seek efficient, green and sustainable business model. Moreover, the innovation and upgrading of independent technologies and the accumulation of high-quality talents accelerate the transformation of industrial structure, virtually optimize the allocation of various factors of production, and reduce the emission of pollutants, which has a great role in promoting the improvement of urban green total factor productivity.

### 4.4. Analysis of Differences among Different Cities 

The resource seeking of FDI and the direction of a country’s macro policy may have an important impact on the industry category and the level of investment, and then affect the efficiency of green economic development. In the following part, the heterogeneous impact of FDI will be tested considering the differential impact of urban resource endowment and national policy orientation.

First of all, compared with non-resource-based cities, resource-based cities are rich in minerals and other resources, which are attractive to resource-seeking FDI. While the soft environment for investment is poor. So, the interaction effect on foreign investment is uncertain. The 284 cities are divided into resource-based cities and non-resource-based cities according to the Plan issued by the State Council in 2013. There are 112 resource-based cities, including Tangshan, Shuangyashan, Baise and Baiyin, and 172 non-resource-based cities, including Beijing, Shanghai and Jinan.

Secondly, according to the traditional view of geographical economics, there is a great regional difference between economically developed regions and economically underdeveloped regions [1]. Compared with coastal cities, non-coastal cities have obvious disadvantages in the quantity and quality of foreign investment introduction, so the location advantage should be taken into account when analyzing the effect of FDI on urban green total factor productivity. There are 43 coastal cities in the sample, including Yantai, Shanghai and Qingdao, and so on. There are 241 prefecture-level cities that are not coastal cities, including Hengshui, Taiyuan and Harbin, etc.

Third, national policy guidance will also have an important impact. High-quality Belt and Road cooperation is an important measure for China to participate in the construction of an inclusive global mechanism system. As an important trade and transportation channel connecting China to the Eurasian continent, the China-Europe Railway Express provides new opportunities for the economic development of cities along the route. By 2018, 43 Chinese cities including Changchun, Zhengzhou and Rizhao had opened China-Europe express trains. This paper takes whether a city is the departure station of China-Europe trains as the classification standard for analysis and inspection.

The results of columns (1) and (2) in Table 9 show that the impact of FDI on GTFP of non-resource-based cities fails the significance test. For resource-based cities, FDI significantly reduces the GTFP, and the regression result is significant at the level of 1%, which proves the existence of the “resource curse” phenomenon in resource-based cities. There may be two reasons for this phenomenon. First, it is common for resource-based cities to over-rely on resource-based industries for economic growth. A large amount of capital is used at a low level and with low efficiency. All of those are not conducive to sustainable economic development. Second, with the improvement of the infrastructure in China, the hard environment for cities to attract foreign capital is continuously strengthened, but the phenomenon of dislocation, offside and absence of the governments occurs frequently in resource-based cities, hindering the process of attracting foreign capital. The mismatch of government guidance will greatly hinder the improvement of GTFP in all cities.

The results in columns (3) and (4) in Table 9 show that FDI reduces the GTFP of non-coastal cities and passes the test at the 10% level. However, for coastal cities, the negative effect of FDI does not pass the significance test. On the one hand, industries in non-coastal cities are mainly labor-intensive and resource-intensive, and the government tends to relax environmental regulations to attract foreign investment. Multinational corporations that invest in non-coastal cities are more inclined to obtain high profits by accelerating the use of natural resources. The inflow of low-quality foreign capital seriously damages the local ecological environment and restrains the green development of the economy. On the other hand, most coastal cities are economically developed cities, which have incomparable advantages over non-coastal cities in terms of R&D expenditure and environmental technology, which can promote the green economic transformation to a certain extent through energy conservation, emission reduction and environmental governance investment.

The results of columns (5) and (6) in Table 9 show that the inflow of FDI significantly reduces urban GTFP regardless of whether the sample cities have opened the China-Europe Railway Express or not. The negative effect is more obvious for the cities that have opened the China-Europe Railway Express, significant at the 10% level. The main reason for this phenomenon may be that China has opened China-Europe Railway express trains for a short period of time, and most of the goods involved are clothing, shoes and hats, household and daily goods, etc. The “forcing effect” of high-quality “product threshold” is not significant. In addition, the homogenization of China-Europe Railway Express routes is serious. In order to attract foreign capital to support the development of this city’s freight train, the government blindly gives large subsidies and policy support, which intensifies the vicious game between governments, inhibits the market competitiveness of China-Europe Railway Express, is not conducive to scientific and technological innovation, and hinders the improvement of GTFP [43].

## 5. Conclusions and Suggestions

In the context of pursuing green and high-quality development, based on the panel data of 284 prefecture-level cities in China from 2004 to 2019, this paper uses the SBM model to measure the green total factor productivity of each city, empirically tests the impact of FDI on green total factor productivity, and tests its mechanism from the perspectives of scientific and technological innovation and industrial structure upgrading. The heterogeneity test was conducted considering resource endowment, geographical location and the guiding influence of macro policies. The main conclusions and policy recommendations are as follows:

### 5.1. Conclusions

Firstly, the results of the full sample test show that FDI has a significant inhibitory effect on the urban green total factor productivity improvement, and the negative impact on the green technology progress index is the main reason, the effect of “pollution paradise” is verified. It is consistent with the research conclusions of Chen et al. [14], D’agostino and Lorena [15], and Wang et al. [16]. This conclusion is still significant after a series of robustness tests such as replacing variables and eliminating outliers. The way of investment before does not completely conform to the sustainable development strategy of our country. It is urgent to improve the quality of foreign investment.

Second, scientific and technological innovation is an important intermediary mechanism for improving urban green total factor productivity, and this mechanism has passed the Sobel test and bootstrap random sampling test, and the conclusion is robust. We should adhere to the development path of high scientific and technological innovation and low pollution emissions. It is consistent with the research hypothesis in this paper. The interaction between FDI and industrial structure upgrading has a positive moderating effect on the improvement of urban green total factor productivity. Consistent with the research conclusions of Hang and Ren [18], the upgrading of industrial structure plays an obvious role in the improvement of urban green total factor productivity, and we need to pay attention to the impact of foreign capital introduction on the upgrading of industrial structure.

Thirdly, the impact of FDI on green total factor productivity is heterogeneous. specifically, the inhibitory effect of FDI on resource-based cities is stronger than that on non-resource-based cities, on China-Europe Railway Express opening cities is stronger than that on non-opening cities, and on coastal cities is stronger than that on non-coastal cities. This is an important conclusion found in this paper according to China’s national conditions, combined with the characteristics of foreign direct investment and green total factor productivity. It is of guiding significance for formulating differentiated urban investment policies and improving the green development level of cities in the low green total factor productivity group. In the context of green economic development, if we want to expand openness and green economic development, our government should further improve the quality of investment, stimulate local innovation, eliminate highly polluting industries and promote the upgrading of industrial structure. Considering the availability of data, this paper mainly uses the data of cities in eastern and central China for research. With the acceleration of the opening-up and development of the western region and the improvement of relevant data, the author will conduct further research and discussion, so as to get a more universal conclusion.

### 5.2. Policy Recommendations

1. Focus on improving the quality of foreign investment, change the assessment method of investment attraction and promote green and high-quality development.

The evaluation method based on the scale of foreign investment cannot meet the needs of the development of new technological revolution and the transformation and upgrading of domestic industries. Under the new pattern of development, the government should adjust and improve the policy of introducing foreign investment. The new evaluation system of attracting foreign investment should focus on combining attracting foreign investment with introducing high-end elements such as core and key technologies, modern management and international talents, improve the screening of foreign investment inflow, and curb the entry of energy-intensive foreign enterprises. In particular, some cities with low development level of green total factor productivity and low foreign direct investment should abandon the traditional development mode of blindly attracting foreign investment regardless of environmental costs, improve the adaptability of environmental regulation and high-quality FDI, refuse the “bottom-by-bottom competition” formed to attract more foreign investment, thoroughly implement the five development concepts, and accelerate the construction of a resource-saving and environmentally friendly society.

2. Accelerate the establishment of technological innovation system, promote the transformation and upgrading of industrial structure and realize the positive influence mechanism of both.

Based on scientific and technological innovation, promote the transformation and upgrading of industrial structure. Coastal cities and regional central cities have a better scientific and technological foundation, strong innovation capacity, and a more optimized industrial structure, which also has a more obvious positive impact on green total factor productivity. Cities with low innovation ability and weak industrial foundation should establish an innovation system with enterprises as the main body, market-oriented and the combination of industry, university and research, improve the original innovation ability, integrated innovation ability and the ability to introduce, digest, absorb and re innovate, drive industrialization with informatization, promote the faster and better development of clean industry. The government’s industrial policy should aim at promoting industrial transformation and actively encourage regional scientific and technological innovation, pay attention to the combination of attracting foreign investment with introducing intelligence, technology and new business forms, encourage foreign investment in big data, emerging areas such as artificial intelligence, speed up the development of the tertiary industry and the transformation of industrial structure. Enterprises are encouraged to use advanced and applicable technologies to improve traditional industries and accelerate the development of green industries and modern service industries. The government actively guides the merger and reorganization of traditional manufacturing industry, improves the system and mechanism of green industry development, and gives full play to the positive role of the two in green total factor productivity.

3. Scientifically implement national strategies, formulate foreign investment introduction policies according to local conditions and improve the green development level of cities in strategic core areas.

The government should strictly implement the national policy requirements, increase strategic input, change the way of strategy implementation and policy guidance, and further improve the quality and level of the implementation of the Belt and Road and other national development strategies. The government should provide differentiated policy support for key development regions, adopt diversified policy schemes of “one place, one policy”, and lead local policies to develop towards diversification and high efficiency. In eastern coastal areas, investment should focus on emerging industries and modern service industries, actively cultivate innovation and entrepreneurship incubation bases. Infrastructure and public services in the central and western regions should be strengthened, and the pooling capacity of capital, personnel and other factors of development should be increased. Cities in strategic core areas seize strategic opportunities such as “The Belt and Road” and “double circulation”, establish inland ports and other ways to promote the linkage between land and sea and realize the inland areas from the tip of opening to the forefront of opening up. Resource-based cities should gradually get rid of resource dependence through industrial transformation, improve the quality of investment attraction and improve the level of green total factor productivity by upgrading the technological level and optimizing the industrial structure.

## Figures and Tables

**Table 1:** Input-output index of green total factor productivity measurement.

Variable	Indicators	Measure	Data Source
Input	Labor	Number of employed persons over the years (ten thousand)	
	Capital	Physical capital stock calculated by the perpetual inventory method (CNY ten thousand)	China City Statistical Yearbook
	Energy	Total annual electricity consumption (10,000 KWH)	
Expect Output	Economic output	Real GDP (CNY billion)	China City Statistical Yearbook
Undesired Output	Environmental pollution	Industrial wastewater discharge (ten thousand tons)	China Environmental Statistics Yearbook
		Sulfur dioxide emissions (ten thousand tons)	
		Industrial dust emission (ten thousand tons)	

**Table 2:** Variable Description.

Variable	Variable Name	Indicator Description	Data Source
Explained Variable	Green total factor productivity (ML)	Green total factor productivity	The authors calculated
Explanatory Variables	Stock of foreign direct investment (FDI)	Actual utilization of foreign capital stock	China City Statistical Yearbook
Control Variables	Resource abundance (RES)	The number of mining employees accounts for the total number of people employed	China City Statistical Yearbook
	Environmental regulation (ER)	Three wastes emission	China Environmental Statistics Yearbook
	Infrastructure construction (INF)	Urban road area per capita	China City Statistical Yearbook
	Degree of government intervention (GOV)	Government fiscal spending accounts for the regional GDP	China City Statistical Yearbook

**Table 3:** Model selection check.

	B-P Test	Hausman Test	Conclusion
Statistical value	6364.34	58.21	Panel fixed effects model
*p* value	0.0000	0.0000	

**Table 4:** Benchmark regression results.

Variable	(1)	(2)	(3)	(4)	(5)
	lnML	lnML	lnML	lnML	lnML
lnFDI_it_	−0.00771 **	−0.00691 **	−0.00931 ***	−0.00806 **	−0.00792 **
	(0.00340)	(0.00339)	(0.00343)	(0.00342)	(0.00341)
lnRES_it_		−0.0113 ***	−0.0115 ***	−0.0115 ***	−0.0115 ***
		(0.00232)	(0.00231)	(0.00230)	(0.00230)
lnER_it_			0.0103 ***	0.0108 ***	0.0106 ***
			(0.00234)	(0.00233)	(0.00233)
lnINF_it_				−0.0514 ***	−0.0514 ***
				(0.00777)	(0.00777)
lnGOV_it_					−0.0149 *
					(0.00857)
CONS	0.946 ***	0.925 ***	0.927 ***	0.959 ***	0.928 ***
	(0.0451)	(0.0451)	(0.0450)	(0.0451)	(0.0483)
Urban Fixed Effect	Control	Control	Control	Control	Control
Time Fixed Effect	Control	Control	Control	Control	Control

Note: * significance level: 10%, ** significance level: 5%, *** significance level: 1%.

**Table 5:** Regression results of decomposition items of green total factor productivity.

Variable	(1)	(2)	(3)	(4)
	lnMEC	lnMEC	lnMTC	lnMTC
lnFDI_it_	0.00147	0.00260	−0.00664 *	−0.00656 *
	(0.00315)	(0.00320)	(0.00355)	(0.00361)
CONS	0.666 ***	0.672 ***	0.955 ***	0.931 ***
	(0.0417)	(0.0452)	(0.0471)	(0.0511)
Control variable	Uncontrolled	Control	Uncontrolled	Control
Urban Fixed Effect	Control	Control	Control	Control
Time Fixed Effect	Control	Control	Control	Control

Note: * significance level: 10%, *** significance level: 1%.

**Table 6:** Robustness test results.

Variable	(1)	(2)	(3)
	Replace Explanatory Variables	Replace Control Variable	Tailing Test
FDI/GDP	−0.439 *		
	(0.240)		
lnFDI_it_		−0.00618 *	−0.00627 *
		(0.00342)	(0.00342)
CONS	1.347 ***	1.219 ***	0.921 ***
	(0.242)	(0.126)	(0.0512)
Control variable	Control	Control	Control
Urban Fixed Effect	Control	Control	Control
Time Fixed Effect	Control	Control	Control

Note: * significance level: 10%, *** significance level: 1%.

**Table 7:** Intermediary effect regression results.

Variable	(1)	(2)	(3)
	lnML	lnRD	lnML
lnFDI_it_	−0.00792 **	0.00241 ***	−0.00845 **
	(0.00341)	(0.000708)	(0.00342)
lnRD_it_			0.218 ***
			(0.0754)
CONS	0.928 ***	0.0345 ***	0.921 ***
	(0.0483)	(0.0100)	(0.0483)
Control variable	Control	Control	Control
Urban Fixed Effect	Control	Control	Control
Time Fixed Effect	Control	Control	Control
Sobel test	Sobel|Z| = 4.625 *p* = 0.00		
bootstrap test	Direct effect interval: [−0.326, −0.149] Indirect effect interval: [−0.069, −0.011]		

Note: ** significance level: 5%, *** significance level: 1%.

**Table 8:** Regression results of regulatory effect.

Variable	(1)	(2)
	lnML	lnML
lnFDI_it_	−0.00792 **	−0.0558 ***
	(0.00341)	(0.00733)
IS_it_		−1.760 ***
		(0.300)
lnFDI_it_ * lnIS_it_		0.165 ***
		(0.0223)
CONS	0.928 ***	1.419 ***
	(0.0483)	(0.101)
Control variable	Control	Control
Urban Fixed Effect	Control	Control
Time Fixed Effect	Control	Control

Note: ** significance level: 5%, *** significance level: 1%.

**Table 9:** Regression results of sub sample test.

Variable	(1)	(2)	(3)	(4)	(5)	(6)
	Non-Resource City	Resource-Based City	Non-Coastal Cities	Coastal City	Non-China-Europe Train Opening Cities	China-Europe Train Opening Cities
	lnML	lnML	lnML	lnML	lnML	lnML
lnFDI_it_	0.00363	−0.0199 ***	−0.00661 *	−0.0121	−0.00216	−0.0453 *
	(0.00551)	(0.00347)	(0.00365)	(0.0106)	(0.00276)	(0.0256)
lnRES_it_	−0.00764 **	−0.0239 ***	−0.0118 ***	−0.00764 *	−0.0111 ***	−0.0157
	(0.00327)	(0.00291)	(0.00264)	(0.00423)	(0.00193)	(0.0108)
lnERi_t_	0.0172 ***	0.000476	0.0129 ***	−0.00450	0.00709 ***	0.0372 ***
	(0.00354)	(0.00246)	(0.00257)	(0.00554)	(0.00199)	(0.0100)
lnINF_it_	−0.0414 ***	−0.0581 ***	−0.0481 ***	−0.0757 ***	−0.0249 ***	−0.233 ***
	(0.0119)	(0.00802)	(0.00834)	(0.0222)	(0.00637)	(0.0406)
lnGOV_it_	−0.0347 ***	0.00610	−0.00956	−0.0288	−0.00355	−0.106 *
	(0.0133)	(0.00879)	(0.00980)	(0.0183)	(0.00698)	(0.0567)
CONS	0.703 ***	1.150 ***	0.900 ***	1.131 ***	0.852 ***	1.491 ***
	(0.0805)	(0.0459)	(0.0507)	(0.162)	(0.0381)	(0.410)
Urban Fixed Effect	Control	Control	Control	Control	Control	Control
Time Fixed Effect	Control	Control	Control	Control	Control	Control

Note: * significance level: 10%, ** significance level: 5%, *** significance level: 1%.

## Data Availability

The datasets generated and/or analyzed during the current study are available from the corresponding author on reasonable request.
